# Animal Models of Tick-Borne Hemorrhagic Fever Viruses

**DOI:** 10.3390/pathogens2020402

**Published:** 2013-05-28

**Authors:** Marko Zivcec, David Safronetz, Heinz Feldmann

**Affiliations:** 1 Department of Medical Microbiology and Infectious Diseases, University of Manitoba, Winnipeg R3E 0J9, Canada; E-Mails: zivcecm@niaid.nih.gov; 2 Laboratory of Virology Division of Intramural Research, National Institute Allergy and Infectious Disease, National Institutes of Health, Rocky Mountain Laboratories, Hamilton 59840, Montana, USA; E-Mail: safronetzd@niaid.nih.gov

**Keywords:** Omsk hemorrhagic fever virus, Kyasanur forest disease virus, severe fever with thrombocytopenia syndrome virus, Crimean-Congo Hemorrhagic fever virus, animal models

## Abstract

Tick-borne hemorrhagic fever viruses (TBHFV) are detected throughout the African and Eurasian continents and are an emerging or re-emerging threat to many nations. Due to the largely sporadic incidences of these severe diseases, information on human cases and research activities in general have been limited. In the past decade, however, novel TBHFVs have emerged and areas of endemicity have expanded. Therefore, the development of countermeasures is of utmost importance in combating TBHFV as elimination of vectors and interrupting enzootic cycles is all but impossible and ecologically questionable. As *in vivo* models are the only way to test efficacy and safety of countermeasures, understanding of the available animal models and the development and refinement of animal models is critical in negating the detrimental impact of TBHFVs on public and animal health.

## 1. Introduction

Tick-borne pathogens are ubiquitously present throughout the world and are significant global health and agricultural concerns. Ticks serve as vectors and/or reservoirs for bacterial and viral pathogens with enzootic life cycles. The enzootic life cycle of many of these pathogens is often understudied, however with increases in agricultural activity and expansion into previously unutilized lands contact between humans, domesticated and wild animals, and ticks is increasing. Increased contact has resulted in recent emergence of several important zoonotic pathogens and in particular novel viruses. Tick-borne viruses (TBVs) account for a significant portion of tick-borne disease burden especially in the Eurasian and African continents.

Many tick species carry TBVs and the clinical disease caused by these viruses vary broadly. Humans are typically incidental dead-end hosts for TBVs and outbreaks caused by TBVs are usually sporadic, however sustained outbreaks of disease have also been reported [1]. TBVs of clinical concern can be separated into two broad categories: those causing neurologic disease and those causing visceral disease often associated with hemorrhagic manifestations. Clinically relevant TBVs causing neurologic disease including tick-borne encephalitis virus (TBEV) while clinically important visceral TBVs include Crimean-Congo hemorrhagic fever virus (CCHFV), Kyasanur forest disease virus (KFDV) and the related Alkhumra or Alkhurma hemorrhagic fever virus (AHFV), Omsk hemorrhagic fever virus (OHFV) and severe fever with thrombocytopenia syndrome virus (SFTSV). Neurologic TBVs, predominantly TBEV, have higher incidence rates and are extensively reviewed elsewhere [2,3,4,5,6,7]. Visceral TBVs or tick-borne hemorrhagic fever viruses (TBHFVs) have a lower but significant impact on human health and are often understudied. Despite the classification of neurologic versus visceral disease causing TBV it is important to note that TBEV has been reported to cause hemorrhagic manifestation in a limited number of cases [8] and visceral TBVs have been reported to occasionally cause neurologic manifestations [9,10,11,12], however the distinction is based around the typical disease presentation in human patients. Due to the sporadic incidence of these severe diseases research, and in many cases clinical descriptions, have been limited. Significant efforts have been made to develop animal models to characterize disease progression, determine correlates of protection and to screen therapeutics and/or vaccines. This review will focus on animal models of TBHVs and to compare disease manifestations to human disease.

## 2. Omsk Hemorrhagic Fever Virus

### 2.1. Epidemiology

During the 1940s, physicians in the Omsk region of Russia noted unusual cases of febrile illness accompanied by hemorrhagic manifestations. Reports of disease were limited to a restricted geographic region and were associated with muskrat hunting and/or processing [13]. The etiologic agent of the disease, named Omsk Hemorrhagic Fever (OHF), was a flavivirus named Omsk hemorrhagic fever virus (OHFV) [9]. Ticks were suspected as vectors of OHFV, which was found to be transmitted to humans by direct contact with infected muskrats or through bite by *Dermacentor reticulatus* or *D. marginatus*. Those ticks are reputably the main vectors of OHFV, and exhibit transovarial and transstadial transmission of OHFV, however gamasid mites and *Ixodes persulcatus* are potentially also involved in OHFV’s sylvatic enzootic cycle [9]. 

**Figure 1:**
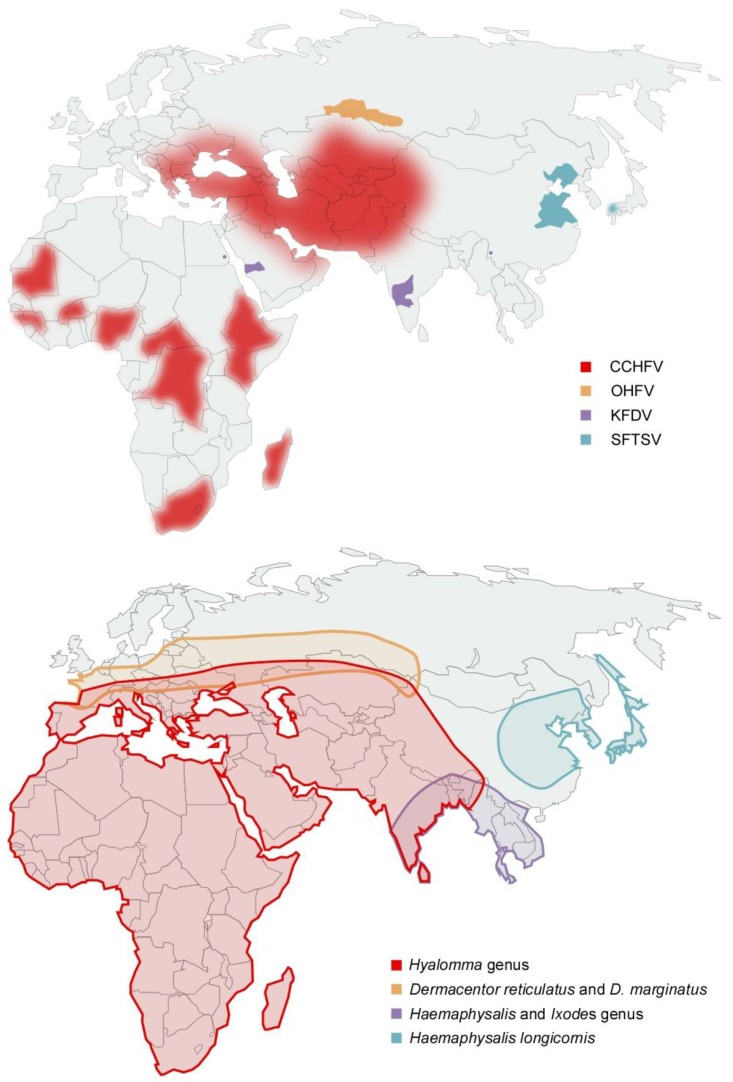
(**a**) Distribution of laboratory confirmed cases of tick-borne hemorrhagic fever (**b**) Distribution of tick species suspected or demonstrated to be vectors of tick-borne hemorrhagic fever viruses. OHFV is transmitted primarily by *Dermacentor reticulatus* and *D. marginatus*; KFDV has been shown to be transmitted by *Haemaphysalis (H. spinigera, H. turturis, H. papuana kinneari, H. minuta, H. cuspidata, H. bispinosa, H. kyasanurensis*, *H. wellingtoni*, *and H. aculeate)*, and *Ixodes (I. petauristae and I. ceylonensis)* genus ticks; SFTSV is suspected to be transmitted by *Haemaphysalis longicornis* and CCHFV is transmitted by *Hyalomma spp* ticks. OHFV = Omsk hemorrhagic fever virus, KFDV = Kyasanur forest disease virus, SFTSV = Severe fever with thrombocytopenia syndrome virus, CCHFV = Crimean-Congo hemorrhagic fever virus.

OHF has been reported in the Russian regions of Kurgan, Tjumen, Omsk and Novosibirsk; however the vectors of disease have a more broad distribution range (Figure 1) [14]. In the first decade following discovery, dozens of OHF patients were reported annually, however incidence of OHF cases has decreased 3- to 5-fold and is thought to be associated with a decrease in muskrat populations and hunting [9,13]. OHF has two seasonal peaks, and corresponds to increases in tick activity and the muskrat hunting season during which the majority of cases are reported [9]. OHF occurs predominantly in young adults (muskrat hunters and spouses involved with muskrat pelt processing) and children under 15 years of age (usually family members of muskrat hunters involved in muskrat pelt processing) [9]. 

### 2.2. Clinical Presentation

Due to the geographically limited nature of the outbreaks clinical data on OHF is limited (Table 1) [9]. However, from the available clinical data OHF begins with an incubation period, which typically lasts between 3–7 days post infection (p.i.). Following the incubation period patients develop a high fever (39–40 °C), cephalalgia, cough and myalgia, often accompanied by dehydration, gastrointestinal (GI) symptoms, facial hyperemia, epistaxis, gingival and vaginal hemorrhage, hemorrhagic rash, thrombocytopenia, neutrophilia and hepatomegaly, for approximately 1–2 weeks. OHF is often biphasic and between 30–50% of patients develop a second phase of disease 1–2 weeks after primary phase resolution [9,15]. The second disease phase manifests with similar symptoms and duration as the first disease phase; however, development of neurological symptoms is more common. The case fatality rates vary by outbreak but are between 0.4%–2.5% [9,15]. 

**Table 1:** Comparison of disease signs between humans and mice following Omsk hemorrhagic fever virus infection.

Species	Human	Mouse
Incubation period	3–7 days (average)	5–8 days
Disease duration	8–14 days (up to 5 weeks in biphasic patients)	9–13 days
Biphasic	Yes	No
Disease signs	Fever, cephalalgia, cough, myalgia, dehydration, gastrointestinal symptoms, facial hyperemia, epistaxis, gingival and vaginal hemorrhage, hemorrhagic rash, hepatomegaly	Weight loss, piloerection, lethargy, hunched posture, conjunctival suffusion, neurological signs
Hematologic changes	Thrombocytopenia, leukopenia, neutrophilia, monocytosis	Leukopenia, neutrophilia
Pathologic changes	Respiratory tract, uterus, liver, GI tract	Brain, thymus, spleen, urinary tract, GI tract
Case fatality	0.4–2.5%	100%

### 2.3. Animal Models of Omsk Hemorrhagic Fever

Following OHFV inoculation several animal species develop asymptomatic viral infection including hamsters, Norway rats, African green monkeys (AGM) and bonnet macaques [9,16]. To date only muskrats and mice have been reported to develop severe disease, therefore mouse models have been the primary model used in OHF studies [9,13,15,16,17,18].

BALB/c and C57BL/6 mice are susceptible to disease following inoculation of OHFV by the intraperitoneal (i.p.) and subcutaneous (s.c.) routes. The incubation period of OHF-like disease in mice is 5–8 days p.i. following either route of infection. At disease onset, infected mice display rapid weight loss, ruffled fur, general malaise, hunched posture and conjunctival suffusion with crusting. In addition, mice develop splenomegaly, splenic hyperplasia, splenic necrosis, necrosis and apoptosis in the cerebellum accompanied by infiltration of mononuclear cells, necrosis and inflammation of the thymus, and hemorrhagic manifestations within brain, liver, urinary/reproductive and GI tract. Infected mice also develop transient leukocytopenia and neutrophilia and increases in total bilirubin, blood urea and globulin levels [17,18,19]. Infectious OHFV is detectable in brain, lung, liver, kidney and spleen of BALB/c mice and OHFV antigen is readily detected in the small intestine [18,19]. Disease signs are accompanied by increases in pro-inflammatory cytokines and chemokines, and splenocyte populations [17,18]. OHFV infection is uniformly lethal in the mouse model with an average time of 9 days p.i. to lethal disease [15]. This mouse model therefore mimics several aspects of severe human OHF (Table 1). 

Neither AGM nor bonnet macaques infected with high dose of OHFV develop disease signs. Bonnet macaques do not display viremia or any pathologic manifestations while AGM support OHFV replication and may transiently display a hemolytic syndrome, accompanied with leukopenia, thrombocytopenic purpura and increases in serum aspartate aminotransferase (AST) and alanine aminotransferase (ALT) without other disease signs [16,20]. Therefore, the AGM model of OHF effectively mimics the mild to moderate disease symptoms of human OHF.

### 2.4. Omsk Hemorrhagic Fever Virus Summary

OHF is a clinically important, but rare disease, affecting a select population within a geographically restricted area. Susceptible mice develop a rapid, inflammatory syndrome, which shares some similarity to the most severe cases of human OHF. Murine models will therefore be useful for evaluating the efficacy of OHF countermeasures such as vaccines and antivirals; however due to the differences between murine and human immune systems the predictive power of this model to define disease progression and correlates of protection may be limited. Therefore, further research is needed to characterize other animal models, especially AGM, which faithfully replicate the typical human disease progression. 

## 3. Kyasanur Forest Disease Virus

### 3.1. Epidemiology

Beginning in 1950s, cases of a novel febrile illness were reported in humans in Shimoga district of the Karnataka (formerly Mysore) state, India [21]. Concurrent to human cases were reports of increased incidence of finding deceased and/or moribund non-human primates (NHPs) in the surrounding forest [21,22]. The source of both human and NHP disease, named Kyasanur forest disease (KFD), was found to be a flavivirus named Kyasanur forest disease virus (KFDV) [21,22,23]. Ticks were implicated as vectors of KFDV due to association of reported cases with seasonal tick activity. Following extensive studies KFDV was isolated from ticks belonging to the *Haemaphysalis* (*H. spinigera*, *H. turturis*, *H. papuana kinneari*, *H. minuta*, *H. cuspidata*, *H. bispinosa*, *H. kyasanurensis*, *H. wellingtoni*, and *H. aculeate*) *Rhipicephalus (R. haemaphysaloides)*, *Hyalomma (H. marginatum issaci)*, *Ornithodoros (O. crosi)*, *Ixodes (I. petauristae* and *I. ceylonensis*), *Argas (A. persicus)*, and *Dermacentor (D. auratus)* genus and experimental transmission of KFDV was demonstrated by members of the *Haemaphysalis* and *Ixodes* genus of ticks [24,25,26,27,28,29,30,31,32,33,34]. 

Despite the distribution of tick vectors, KFD cases are geographically restricted to Karnataka state and following its discovery annual epidemics, comprising of hundreds of cases, have been documented (Figure 1) [10,14]. The majority of cases are reported during the first 5 months of the year, which is concurrent with greatest tick and human outdoor activity [35]. In 1989 KFDV was isolated from a single febrile patient in China however further analysis suggested that this isolate may have been a lab contaminant as it is nearly identical to the 1957 Indian reference strain P-9605 despite being isolated at a much later date in a geographically distinct area [36,37]; in the 1990s the “KFDV-like” virus AHFV emerged in Saudi Arabia and has been subsequently reported in Egypt (Figure 1a) [38,39,40]. Due to low numbers of case reports (<200 reported) little information about AHFV is available. Unlike KFDV, both mosquitoes and ticks are suspected as vectors due to a lack of history of tick bite in several patients [38,41,42]. Genetic analyses reveal that AHFV may have diverged from KFDV approximately 700 years ago, and is still not considered a virus that is distinct from KFDV by the International Committee on Taxonomy of Viruses [43,44]. 

### 3.2. Clinical Presentation

KFD begins with an incubation period estimated to be between 3 and 8 days followed by sudden onset of fever, cephalalgia, diarrhea and severe myalgia (Table 2). KFD lasts approximately two weeks and can include cough, hepatomegaly, splenomegaly, epistaxis, oral and intestinal hemorrhage, and meningoencephalitis. Elevated liver enzymes, thrombocytopenia, leukopenia and anemia are also seen in patients. KFD is often biphasic with apparent convalescence often followed by a second febrile period, which is associated with meningoencephalitis [10,23]. Case fatality rates of 2–10% have been reported for KFD.

**Table 2:** Comparison of disease signs between humans, mice and bonnet macaques following Kyasanur forest disease virus infection.

Species	Human	Bonnet Macaque	Mouse
Incubation period	3–8 days	3 days	3–5 days
Disease duration	8–12 days	3–8 days	6–8 days
Biphasic	Yes	No	No
Disease signs	Fever, cephalalgia, myalgia, diarrhea, cough, hepatomegaly, splenomegaly, epistaxis, bradycardia, oral and intestinal hemorrhage, meningoencephalitis	Fever, diarrhea, bradycardia, hypotension	Paralysis, weight loss
Hematologic changes	Leukopenia, thrombocytopenia, neutropenia, eosinopenia, elevated liver enzymes	thrombocytopenia, leukopenia, elevated liver enzymes	Not reported
Pathologic changes	Kidney, liver, lung	Kidney, liver, lung, spleen and lymph nodes	Brain, lungs
Case fatality	2–10%	100%	≥97%

Clinical presentations of AHF are reported as hemorrhagic manifestations accompanied by liver dysfunction with a case fatality of up to 25% [38,45]. AHF, therefore, mimics severe KFD; however, this observation may be due to lack of reports of mild AHF cases. 

### 3.3. Animal Models of Kyasanur Forest Disease

To date no animal studies have been conducted with AHFV, however based on limited serological data sheep, goats and camels are involved in the transmission cycle of AHFV [42,45].

Several rat and mouse species, gerbil, porcupines, shrews, squirrels, cattle and NHPs (bonnet macaques) have been experimentally infected with KFDV [16,22,46,47,48,49,50,51,52,53,54]. Of the species tested, severe disease was seen in rodents, squirrels and bonnet macaque with pathological investigations carried out only in mice and NHPs.

#### 3.3.1. Rodent Models of Kyasanur Forest Disease

KFDV was first isolated by intracranial (i.c.) inoculation of serum or tissue homogenates from deceased NHPs into newborn (2–3 day old) mice [21]. Further experiments revealed that both young (3–4 week old) and adult (≥5 week old) mice infected by the s.c., i.p. and intranasal (i.n.) route developed a rapid and lethal disease [55,56,57]. The disease course is dependent on both age of inoculated mice and route of infection with time to terminal disease between 5 to 11 days p.i. [57]. Mice develop moderate viremia and significant organ viral loads, especially in the brain [57]. Pathological changes following infection are limited to encephalitis and brain necrosis accompanied by interstitial pneumonitis and hemorrhage in the lungs [57]. Approximately 2–3% of mice survive KFDV challenge and exhibit resistance to subsequent KFDV back challenge [58]. These mice exhibit persistent neurological signs and KFDV can be isolated from these mice up to eight months p.i. [58]. Due to a lack of liver and spleen pathology, presence of significant brain pathology and a rapid, lethal disease course, the mouse model may not be a predictive model of human disease (Table 2); however, it has been utilized to evaluate the protective efficacy of KFDV vaccines [59,60]. 

#### 3.3.2. Non-human Primate Models of Kyasanur Forest Disease

NHPs, specifically bonnet macaques (*Macaca radiata*), are susceptible to lethal KFD; however, the case fatality and disease burden among naturally infected bonnet macaques is unknown. KFDV infected moribund or recently deceased bonnet macaques collected in Kyasanur forest displayed liver and kidney pathology, sometimes accompanied by evidence of encephalitis [22].

Experimentally infected bonnet macaques develop diarrhea, bradycardia, hypotension, thrombocytopenia, leukopenia, anemia and elevated liver enzymes similar to humans [16,54]. During the disease course bonnet macaques develop systemic viral distribution and shedding from pharyngeal surfaces and sometimes in urine [16]. Bonnet macaques are reported to develop either an encephalitic disease or a visceral disease following KFDV infection. The initial study detected lymphoid hyperplasia, non-specific necrosis within liver and kidney, and disseminated non-supportive encephalomyelitis [54]. Another study determined that following KFDV infection bonnet macaques manifest changes in fatty deposition in the liver, depletion of lymphocytes sometimes accompanied by necrosis in the lymphoid organs, and loss of architecture in the GI tract without neurologic involvement [16]. Taken together these data indicate that *M. radiata* develop a disease that mimics severe human disease suggesting that this model may be predictive of human KFD (Table 2).

### 3.4. Kyasanur Forest Disease Summary

KFD is a serious human illness with a limited geographic distribution. It has yearly incidence, which corresponds to peak activity of its tick host. Unlike many TBVs, KFDV has both rodent and NHP models. Although mice do not recapitulate human disease they are effective as screening models for evaluating vaccines and antivirals, while bonnet macaques more accurately recapitulate human disease. 

## 4. Severe Fever with Thrombocytopenia Syndrome Virus

### 4.1. Epidemiology

In 2007, in the Huaiyangshan mountain range in China, patients with an unknown syndrome were reported. The disease was named severe fever with thrombocytopenia syndrome (SFTS) and was found to be a caused by a Phlebovirus of the *Bunyaviridae* family, named severe fever with thrombocytopenia syndrome virus (SFTSV) [61,62,63]. Transmission of SFTSV has not been established experimentally however SFTSV was isolated from pools of and SFTS cases are reported within the geographic distribution of *Haemaphysalis longicornis* implicating this tick species as a viral vector (Figure 1) [14,61,62].

As of early 2013, SFTS cases have been identified in Henan, Hubei, Shandong, Anhui, Zhejiang, Jiangsu, and Liaoning provinces of China and in South-West Japan (Figure 1a) [61,62,63,64,65]. Hundreds of SFTS cases are reported annually and the disease is reported predominantly between the months of May and July, which corresponds to the greatest tick and farming activity [61,62]. In addition to the suspected tick vector, human-to-human transmission due to blood contact has been documented in SFTS cases [66,67,68]. 

### 4.2. Clinical Presentation

The incubation period of SFTS following tick bite or unprotected contact with the blood of a viremic patient is unknown but secondary cases develop symptoms within 3 weeks of contact with a viremic SFTS patient (Table 3) [66,67,68]. SFTS begins with a non-specific prodrome including fever, cephalalgia, myalgia, arthralgia, dizziness and malaise, which persists for 3–7 days [12,68,69,70]. Following the prodrome patients often develop mucosal hemorrhage, hemorrhagic rash, thrombocytopenia, elevated liver enzymes, multi-organ failure, disseminated intravascular coagulopathy (DIC), and central nervous system symptoms such as confusion. The case fatality rate was initially much higher, but currently is 12–15% [12,61,62,68,69,70]. Symptom severity is correlated to immune suppression and viremia level with fatal cases often undergoing immune-suppressive therapy and exhibiting sustained high viremia [12,62,70].

### 4.3. Rodent Models of Severe Fever with Thrombocytopenia Syndrome

SFTSV infection and disease progression has only been described in rodent models, however serological analysis implicates a role of cows, goats, sheep, dogs, pigs, chickens and hedgehogs in the SFTSV transmission cycle [71,72]. 

Adult BALB/c mice, Wistar rats, Kunming (KM) mice and both adult and newborn Syrian hamsters are susceptible to, but do not develop disease following, SFTSV inoculation by the i.p. or i.c. route [73]. However, newborn mice and rats are susceptible to disease following SFTSV inoculation [73].

Following i.c. inoculation of SFTSV newborn Wistar rats, KM, BALB/c, and C57BL/6 mice develop a severe, uniformly lethal disease (Table 3) [73]. The i.p. route of infection decreases the case fatality within susceptible rodents from 100% to 35–50%. SFTS-like disease in newborn KM mice lasts between 7–17 days, depending on the inoculation dose, and is characterized by decrease in body weight and mobility, piloerection and paralysis of the hind limbs. Aside from overt signs of disease newborn KM mice develop necrosis and mononuclear cell infiltration in liver and brains but not in other tissues [73]. 

**Table 3:** Comparison of disease signs between humans and mice following severe fever with thrombocytopenia syndrome virus infection.

Species	Human	Mouse		Suckling Mouse
		Untreated	Mitomycin C treated	
Incubation period	≤21 days	≤3 days	Not reported	6–9 days
Disease duration	3–7 days	≤18 days	≤21 days	2–8 days
Biphasic	No	No	No	No
Disease signs	Fever, cephalalgia, myalgia, arthralgia, dizziness, malaise, mucosal hemorrhage, hemorrhagic rash, multi-organ failure, disseminated intravascular coagulopathy, neurological symptoms	None	Weight Loss	Weight loss, piloerection, lethargy and hind limb paralysis
Hematologic changes	Leukocytopenia, thrombocytopenia, elevated liver enzymes	Leukocytopenia, thrombocytopenia, elevated liver enzymes	Unknown	Elevated total bilirubin, decrease in albumin
Pathologic changes	Unknown	Liver, spleen	Unknown	Liver
Case fatality	12%	0%	50%	35–100%

A non-lethal C57BL/6 mouse model recapitulates some hematological signs of human SFTS (Table 3) [74]. C57BL/6 mice are susceptible to intravenous (i.v.), i.c., i.p., and intramuscular (i.m.) routes of infection by SFTSV and develop a mild, self-limiting disease. This disease is characterized by an acute (<7 days) viremia accompanied by virus replication in kidney and spleen, transient thrombocytopenia, leukocytopenia, and elevated AST. Following decreased viral loads, increases in blood urea nitrogen (BUN) and ALT are observed followed by appearance of transient liver necrosis and impaired renal capsules accompanied by an increase in SFTSV specific cellular and humoral (neutralizing and total IgG) responses. Inhibition of mouse adaptive immune responses with mitomycin C, to mimic immune-suppression in humans, results in a more serious disease during which mice lose weight and have a case fatality rate of 50% [74]. 

Together the rodent data suggest that impairments in the immune system are required for development of severe disease as has been suggested to occur in severe human SFTS [74]. Based on available data rodent models would be useful as *in vivo* screening models for antivirals and vaccines; however, due to the limited work reported, whether these animal models faithfully reproduce human disease remains an open question. 

### 4.4. Severe Fever with Thrombocytopenia Syndrome Virus Summary

SFTS represents a novel viral hemorrhagic fever, which is currently geographically restricted to North-East China and South-West Japan but threatens a large population. However, lack of information on transmission, disease and endemicity, coupled with the potential of person-to-person transmission may allow SFTSV to spread and cause outbreaks even in distant geographic areas e.g. travel. Therefore, the development of effective postexposure treatments is imperative. To this end, existing rodent animal models of SFTS may be useful as screening models for vaccine development and testing of antivirals. However, rodent models do not mimic human disease progression or case fatality and therefore development of alternate animal models, ideally NHP models, would be desirable for further elucidation of SFTSV pathogenesis. 

## 5. Crimean-Congo Hemorrhagic Fever Virus

### 5.1. Epidemiology

In the 1940s, soldiers in Crimea experienced a febrile illness accompanied by severe hemorrhagic manifestations [75,76,77,78]. In the 1950s, another febrile illness accompanied by hemorrhagic manifestations was detected in the Belgian Congo (currently Democratic Republic of Congo). In the late 1960 the etiologic agents of the two illnesses were found to be serologically related [79,80,81] and following further analysis the two were found to be different strains of the same virus. Ultimately, the disease was named Crimean-Congo hemorrhagic fever (CCHF) and the etiologic agent CCHF virus (CCFHV). 

In contrast to other TBHFVs, CCHFV is widely distributed and has been reported in over 30 countries spanning Africa, South-Eastern Europe, the Middle East and Western Asia (Figure 1a) [75,76,77,78,82]. The incidence of disease is associated with the distribution and activity of its vectors, *Hyalomma* genus ticks, and transmission of CCHFV to mammals has been demonstrated for several *Hyalomma* members (Figure 1b) [14,83,84,85,86,87]. The predominant vector of CCHFV varies geographically and include *H. anatolicum* subspecies (especially *H anatolicum anatolicum*) which are distributed throughout Eurasia and the northern half of Africa; *H. marginatum* subspecies (*H. marginatum marginatum*, *H. marginatum rufipes*, *H. marginatum turanicum* and *H. marginatum isaaci*) which are distributed throughout Eurasia and Africa; *H. truncatum*, *H. impeltatum*, *and H. impressum* which are distributed primarily within Africa [88]. Since its discovery, hundreds of CCHF cases were reported annually; however, recent outbreaks of CCHF have become more frequent with thousands of case reports annually and the endemic area has expanded to include additional countries within Eurasia.

CCHFV has a wide host range and can cause a transient viremia in many wild, domesticated and laboratory mammals [75,76,78,89,90], but infection is refractory in most birds [84,91]. CCHFV is transmitted to humans by bite of an infected tick, crushing an engorged infected tick, or by contact with body fluids of viremic humans or animals, suggesting CCHFV is capable of infecting humans via multiple routes of infection.

### 5.2. Clinical Presentation

Following the incubation period, typically 1 to 7 days post exposure, patients experience a rapid onset of high grade fever, fatigue, cephalalgia, dizziness, photophobia and myalgia, often with nausea, vomiting and diarrhea (Table 4) [75,76,78]. Hemorrhagic manifestations develop in severe cases, typically lasting two to three days and are characterized by thrombocytopenia, increases in liver enzymes, petechiae, ecchymosis, epistaxis, gingival hemorrhage, often accompanied by GI and cerebral hemorrhages. Convalescence typically starts 10 to 12 days post symptom onset. The case fatality is variable and ranges from approximately 5% in Turkey [92], to ~60% in the 1994–1995 CCHF outbreak in the United Arab Emirates [93,94,95], but is more often reported to be approximately 30% [75,76,78]. Predictors of fatal outcome in human CCHF are high viral loads, increased serum AST and ALT, severe thrombocytopenia, increased clotting times, increased serum levels pro-inflammatory cytokines and chemokines, low antibody titers and presence of melena [96,97,98,99,100,101,102,103,104,105,106,107,108]. Severe liver pathology, DIC, shock and multiorgan failure are the main causes of death [75,76,78,109]. 

**Table 4:** Comparison of disease signs between humans and mice following Crimean-Congo hemorrhagic fever virus infection.

Species	Human	Newborn mice	IFNAR^−/−^ mice	STAT1^−/−^ mice
Incubation period	1–7 days	≤3 days	≤3 days	≤2 days
Disease duration	10–12 days (average)	4–5 days	2–3 days	1–2 days
Biphasic	No	No	No	No
Disease signs	Fever, cephalalgia, myalgia, arthralgia, dizziness, malaise, mucosal hemorrhage, hemorrhagic rash, multi-organ failure, disseminated intravascular coagulopathy	Weight loss, piloerection, lethargy, hind limb paralysis	Weight loss, piloerection, lethargy, occasional hemorrhage	Weight loss, piloerection, lethargy, occasional hemorrhage
Hematologic changes	Leukocytopenia, thrombocytopenia, increase clotting times, increased pro-inflammatory cytokines/chemokines, elevated liver enzymes	Unknown	Thrombocytopenia, increased clotting times, elevated liver enzymes, increased pro-inflammatory cytokines/chemokines	Thrombocytopenia, elevated liver enzymes, increased pro-inflammatory cytokines/chemokines
Pathologic changes	Liver, spleen, lung		Liver, spleen, lymph node	Liver, spleen
Case fatality	3–60%	100%	100%	100%

### 5.3. Animal Models of Crimean-Congo Hemorrhagic Fever

Adult mice, rats, hamsters, guinea pigs, rabbits, ostriches, cattle, sheep, goats, donkeys, horses, and NHPs are susceptible to CCHFV infection but do not develop signs of disease [91,110,111,112].

Newborn mice and rats are susceptible to infection by the i.p. and i.c. routes and exhibit a uniformly fatal disease with neurological signs, which are uncommon in human cases (Table 4) [110]. Newborn mice exhibit weight loss, and paralysis accompanied by viremia and high viral organ titers with an average time to lethal disease of eight days p.i. [110,113]. The only adult animal models available for CCHFV are mouse strains deficient in type I Interferon signaling [114,115,116].

Signal transduction and activator of signaling-1 knockout (STAT1^−/−^) and Interferon αβ receptor knockout (IFNAR^−/−^) mice are susceptible to lethal disease following CCHFV infection [114,115,116]. Following infection, both STAT1^−/−^ and IFNAR^−/−^ mice exhibit a rapid (4–6 days), uniformly lethal disease which mimics several aspects of severe human disease. Both mouse strains exhibit piloerection, lethargy, hunched posture and weight loss accompanied by hemorrhagic manifestations [114,115,116]. In addition, both mouse strains develop thrombocytopenia and liver dysfunction. Virus is detected in all tissues accompanied by viremia. Pathological changes include severe necrosis and mononuclear cell infiltration of the liver, and lymphocytolysis and necrosis in spleen and lymph nodes. Pathological changes are associated with high levels of viral antigen. Virus infection causes increases in pro-inflammatory chemokines and cytokines suggesting inflammatory responses contribute to disease progression. Therefore, the STAT1^−/−^ and IFNAR^−/−^ mouse CCHF models mimic several aspects of severe human disease (Table 4) [114,115,116]. However, these mice do not recapitulate human disease progression and are therefore likely going to be most useful as screening models for CCHFV countermeasures.

### 5.4. Crimean-Congo Hemorrhagic Fever *virus* Summary

CCHFV is the most widely distributed TBHFV with an endemic area of >30 countries. CCHFV infects a wide range of mammalian hosts but only causes disease in humans, newborn mice and mice strains deficient in type I interferon signaling. While knockout mouse strains offer a tool to assess CCHFV countermeasures, disease progression in these animals differs from human CCHF, which limits the use of the models to study CCHF disease progression. Therefore, the development of animal models, ideally NHP models, which more faithfully reproduce human clinical manifestations are needed to assess disease progression and to confirm efficacy of CCHFV countermeasures *in vivo*.

## 6. Conclusions

TBHFV are present throughout the Eurasian and African continents and are an emerging or re-emerging threat to many nations. To date, with the exception of CCHFV, TBHFV are geographically limited and not well studied. As a result, limited research tools, most notably animal models, are available for most of these pathogens. In the past decade, however, TBHFVs have emerged (*i.e.*, SFTSV) and areas of endemicity have expanded (*i.e.*, CCHFV and KFDV). Therefore, it is critical to develop new and refine existing animal models for TBHFVs, which mimic human disease, ideally NHPs, to be prepared for further escalation in disease burden. NHP models are preferred as national regulatory authorities, such as the US Food and Drug Administration, consider NHP models the gold standard for efficacy testing of drugs and vaccines for pathogens that would be unethical or unfeasible to be tested in humans [117]. Development of countermeasures is of utmost importance in combating tick-borne viruses as elimination of vectors and interrupting enzootic cycles is all but impossible and ecologically questionable. As *in vivo* models are the only way to test efficacy and safety of countermeasures, development and refinement of animal models is critical in negating the increasing detrimental impact of TBHFVs on public and animal health. 
